# Utilization of IκB–EGFP Chimeric Gene as an Indicator to Identify Microbial Metabolites with NF-κB Inhibitor Activity

**DOI:** 10.1007/s12575-010-9033-9

**Published:** 2010-10-02

**Authors:** Yu-Ling Lin, Yen-Shun Chen, Jui-Hung Hsieh, Ching-Min Lin, Hsin-Yi Wu, Chen-Si Lin, Rea-Min Chu, Kuang-Wen Liao, Yuan-Hsun Hsu

**Affiliations:** 1 Institute of Molecular Medicine and Bioengineering, National Chiao Tung University, Hsinchu, Taiwan; 2 Graduate Institute of Medical Sciences, Taipei Medical University, Taipei, Taiwan; 3 Department of Electronics Engineering and Institute of Electronics, National Chiao Tung University, Hsinchu, Taiwan; 4 Animal Cancer Center, School of Veterinary Medicine, National Taiwan University, Taipei, Taiwan; 5 Department of Biological Science and Technology, National Chiao Tung University, 300 Room 205 Zhu-Ming Building, 75 Bo-Ai Street, Hsinchu, Taiwan; 6 Department of Microbiology and Immunology, College of Medicine, Taipei Medical University, Taipei, Taiwan

**Keywords:** Microbial metabolite, Antioxidant, IκB, EGFP, Hydroquinone, Propolis

## Abstract

NF-κB regulates several important expressions, such as cytokine release, anti-apoptosis, adhesion molecule expression, and cell cycle processing. Several NF-κB inhibitors have been discovered as an anti-tumor or anti-inflammatory drug. The activity of NF-κB transcription factor is negatively regulated by IκB binding. In this study, IκB assay system was established and IκB–EGFP fusion protein was used as an indicator to monitor the effects of substances on the IκB degradation. The results indicated that the chosen hydroquinone could inhibit the IκB degradation and cause the cell de-attachment from the bottom of culture plate. In addition, this system could also monitor the IκB degradation of microbial metabolite of natural mixtures of propolis. Thus, the IκB assay system may be a good system for drug discovery related to microbial metabolite.

## 1 Introduction

NF-κB is an inducible transcription factor involved in the regulation of gene expression for cytokine release, anti-apoptosis, adhesion molecule, and cell cycle regulation [1]. There are five members in the NF-κB family composed of p50/p105, p52/p100, c-Rel, Rel A, and Rel B [2]. These components all have a consensus amino acid domain that is Rel homology domain (RHD). RHD, a regulatory element for activity of NF-κB, is involved in NF-κB dimerization and IκB binding. Without intracellular stimulation, IκB associates with RHD of NF-κB to inhibit NF-κB migration into the nucleus and retain NF-κB in cytosol. As extracellular signals of NF-κB activation transduce into cells, IκB will be phosphorylated and results in degradation. The degradation of IκB can abolish the inhibitory effect on the activity of NF-κB. Therefore, the amount of intracellular IκB can be an indicator for the activity of NF-κB.

The microbial metabolite is an important source of certain medical reagents. Particularly, many investigators indicated that microbial metabolites provide a source of abundant antioxidants. Previous investigations revealed that antioxidants have the ability to inhibit activity of NF-κB [3-5]. Antioxidants, such as β-catenin and bortezomib, have been reported to have a cytotoxic ability against tumor cells by inhibiting the activity of NF-κB [6,7]. Therefore, utilizing the principle of NF-κB activity regulation to develop a drug discovery system may help to rapidly find new drugs.

In this study, we have focused on the establishment of a rapid and precise screening system with NF-κB inhibitory activity, and the metabolite products of microbes and the natural extract propolis were tested in this system. The plasmid pIκB–EGFP, which contained a chimeric gene that encodes a fusion protein, green fluorescent protein (EGFP) fused with IκB-α (IκB–EGFP), was used to establish the screening system. The pIκB–EGFP was transfected into cells and the transfected cells were treated with microbe-derived antioxidants or propolis, and then the fluorescence intensities of IκB–EGFP in the transfected cells were determined by flow cytometry. In our system, if the drug candidates have the activity against IκB degradation, they would protect the IκB–EGFP from degradation and would result in an increase in fluorescence level in the transfected cells. Therefore, the IκB–EGFP proteins were used as an indicator for the degree of IκB degradation to reflect the level of NF-κB activation in the cell. In the screening program, we found that the pure chemical microbe-derived hydroquinone (HQ) and certain propolis obtained from Brazil have significant activities against IκB degradation. Furthermore, HQ was studied and showed that it could affect cell adhesion activity and that the feature is exactly associated with NF-κB activity except for its anti-IκB degradation activity. In conclusion, this study showed that the drug discovery system can exactly identify the effective substance with anti-IκB degradation ability no matter whether the effective substance exists as a pure chemical compound or as a natural mixture. Our data reveals that the transgenic cells can be a convenient, efficient, and exact tool to rapidly identify a new drug for improvement of human health.

## 2 Materials and Methods

### 2.1 Reagents

Plasmid pIκB–EGFP was purchased from BD Biosciences Clontech (CA, USA). Rabbit anti-human IκB-α and rabbit anti-phosphorylated IκB-α polyclonal IgGs were purchased from Calbiochem-Novabiochem Corporation (CA, USA). Hydroquinone (HQ), homogentisic acid (HA), protocatechuic acid (PA), and β-phenylpyruvic acid (β-PPA) were previously isolated from fermentation broth of *Streptomyces* sp. in our laboratory as an antioxidant. The antioxidant curcumin is a well-known inhibitor for IκB degradation and was purchased from Sigma Corporation (St Louis, MO, USA).

### 2.2 Cell Lines and Cell Culture

The K-BALB (murine sarcoma virus-transformed BALB/3T3), 293 (human fetus kidney cells), Hep3B (human hepatocellular carcinoma), Daudi (human Burkitt's lymphoma), and COLO320 (human colorectal adenocarcinoma) were kindly provided by Dr. Steve R. Roffler, Academia Sinica, Taipei, Taiwan. K-BALB, 293, and Hep3B were grown in Dulbecco's modified Eagle's medium (DMEM) supplemented with 10% fetal bovine serum (FBS), penicillin (100 U/ml), and streptomycin (100 U/ml) (DMEM complete media) at 37°C in a humidified 5% CO_2_ atmosphere. Daudi and COLO320 cells were propagated in RPMI 1640 medium with 2 mM L-glutamine adjusted to contain 1.5 g/l sodium bicarbonate, 4.5 g/l glucose, 10 mM HEPES, 1 mM sodium pyruvate, 10% FBS, penicillin (100 U/ml), and streptomycin (100 U/ml). Huh7 cell line was kindly provided by Dr. Chao-Lin Huang, Institute of Preventive Medicine, Taipei, Taiwan, and was grown in DMEM supplemented with 10% FBS, penicillin (100 U/ml), and streptomycin (100 U/ml).

### 2.3 Transgene Expression and Microbial Metabolite-Derived Antioxidant Activity Measurement

Before transfection, 2.5 × 10^5^ Huh7 or 293 cells per well were cultured overnight in six-well plates. Cells were typically ~70% confluent at the time of the experiment. In all transfection assays, 200 μl/well of the transfection sample containing 3 μg plasmid DNA (pIκB–EGFP) and 10 μl Lipofectamine (Invitrogen, NY, USA) in Opti-MEM (Invitrogen) were added and incubated with the cells. The 2 ml/well serum containing DMEM complete media was added after 16 h, and gene-transfected cells were re-suspended and divided equally into each well of a 24-well plate. The 24-h incubation was then performed and 100 μM of each antioxidant, HQ, HA, PA, and β-PPA was administered for 24 h on the transfectants. Next, 50 ng/ml of phorbol 12-myristate 13-acetate (PMA) (Sigma) was added after 2-h incubation to induce IκB–EGFP degradation. IκB–EGFP reporter gene expression was assayed with flow cytometry (BD Biosciences Clontech). Assays were performed in triplicate.

### 2.4 Propolis Preparation and Application into the IκB–EGFP Drug Discovery System

Taiwan propolis (propolis 1 and 2), Brazil red propolis (propolis 3 and 4), and Brazil green propolis (propolis 5 and 6) were provided by Dr. Rea-Min Chu's laboratory and dissolved in 80% ethanol. Then 2.5 × 10^5^ of 293 cells per well were transfected with 3 μg pIκB–EGFP. After 24-h incubation, 1 g/ml of different propolis sources described above, ethanol (80%), and curcumin (10 μM) were added into transfected cells. After 2-h incubation, pIκB–EGFP-transfected 293 cells were treated with 50 ng/ml PMA to initiate IκB–EGFP degradation. Curcumin was used as positive control for its ability to inhibit IκB decomposition. IκB–EGFP reporter gene expression was assayed by flow cytometry after 24-h incubation of propolis.

### 2.5 Western Blot for IκB Expression

After HQ treatment (0–100 μM) for 48 h, pIκB–EGFP-transfected 293 cells were harvested. For immunoblotting, equivalent amounts of cell lysate were resolved by SDS–PAGE (10%) and transferred onto PVDF membranes. After blocking, the membranes were incubated with the anti-IκB and anti-phosphorylated IκB antibody (Santa Cruz Biotech, Heidelberg, Germany). The membranes were then treated with goat anti-rabbit peroxidase-conjugated antibody, and the immunoreactive proteins were detected using enhanced chemiluminescence kits (Pierce, Rockford, IL, USA) according to the manufacturer's instructions.

### 2.6 293 Cells Detachment Assay

293 Cells (2.5 × 10^5^/well) were seeded in a six-well plate and cultured overnight at 37°C in a humidified 5% CO_2_ atmosphere. After overnight incubation, low dosage of HQ (10 μM) was added into each well and the cell growth pattern was observed (0–4 h).

### 2.7 Cell Cytotoxicity Assay

HQ cytotoxicity against COLO320, Daudi, K-BALB, and Hep3B cell lines was determined via MTS assay (CellTiter 96^®^ AQ_ueous_ One Solution Cell Proliferation Assay; Promega, WI, USA). For each cell line tested, a 96-well microtiter plate was prepared by adding 100 μl of cells suspended in growth medium at a concentration of 2 × 10^5^ cells/100 μl/well to each experimental and control well. One hundred microliters of growth medium containing different concentrations of HQ was added to the experimental wells. Another growth medium (200 μl) was added to one column to serve as a blank. After 48-h incubation at 37°C under a water-saturated sterile atmosphere with 5% CO_2_, the medium was removed, and then fresh growth medium (100 μl) and MTS (20 μl/well) were applied to each well for a 4-h incubation. The absorbance of each well at 450 nm was determined by analysis with an ELISA reader (TECAN, Austria). LD50 was defined as the dosage of HQ that produces death in 50% (calculated from the absorbance) of the exposed cell population.

## 3 Results

### 3.1 Antioxidants Derived from Microbial Metabolites were Selected for Anti-IκB Degradation Activity with the IκB–EGFP Drug Discovery System

**Figure 1:**
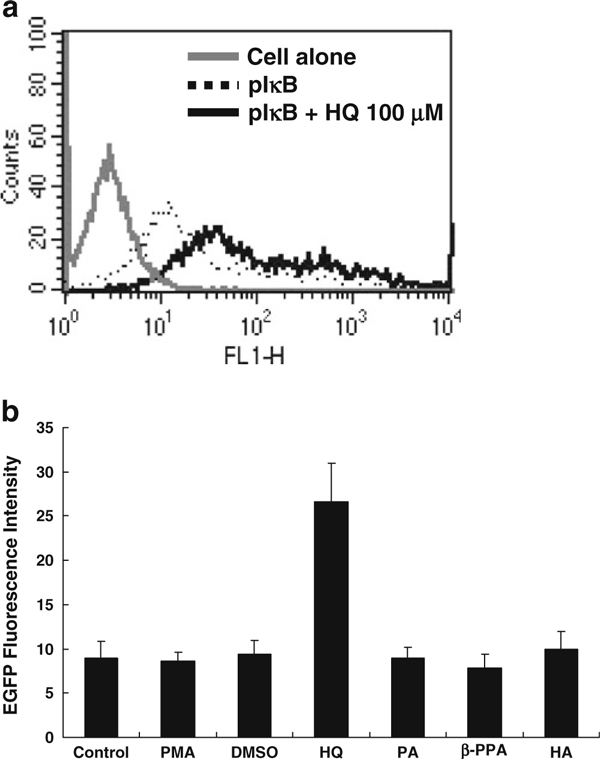
**The accumulation of IκB–EGFP proteins in pIκB–EGFP-transfected Huh7 cells caused by microbial metabolite-derived antioxidant**. **a** The representative histogram pattern of pIκB–EGFP-transfected cell with or without antioxidant treatment. **b** Antioxidants including HQ, PA, β-PPA, and HA (100 μM) were added into pIκB–EGFP-transfected Huh 7 cells. Growth medium was also added into another well as control. After 24-h incubation of the antioxidants, the amount of IκB–EGFP proteins (shown as fluorescence intensity) was measured by flow cytometry (results expressed as mean ± standard deviation).

The pIκB–EGFP plasmid was transfected into Huh 7 cells, and the genetically modified cells were used as an indicator to disclose the candidates with the activity against IκB degradation as NF-κB inhibitors. These antioxidants, such as HQ, PA, β-PPA, and HA, were further determined whether they had anti-IκB degradation activities. The genetically modified cells were incubated with the microbe-derived antioxidants (100 μM), and later PMA (50 ng/ml) was added to accelerate the process of IκB degradation. After incubation, the fluorescence intensities of the genetically modified Huh 7 cells were determined by flow cytometry. The results showed that the addition of HQ caused accumulated IκB–EGFP proteins in the cytoplasm of the transfectant cells to result in 3-fold higher fluorescence intensities compared with the untreated transfected cells, whereas the others could not affect the fluorescence intensities (Figure 1). Therefore, HQ may have the activity to inhibit the IκB degradation.

**Figure 2:**
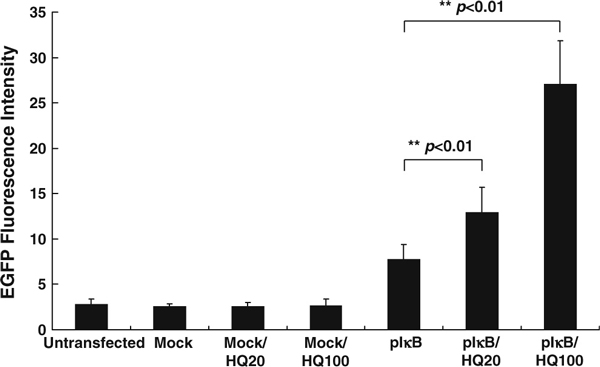
**Fluorescence intensity induced by HQ-treated pIκB–EGFP-transfected Huh 7 cells.** Mock- or pIκB–EGFP-transfected Huh 7 cells were treated with 20 and 100 μM HQ. After 24-h incubation, the amount of IκB–EGFP proteins (shown as fluorescence intensity) was measured by flow cytometry (results expressed as mean ± standard deviation).

Although we found that the fluorescence intensity of pIκB–EGFP transfectant cells is increased after the addition of HQ, many chemicals can be induced to emit fluorescence by the laser beam of a flow cytometer. Therefore, HQ was determined whether the fluorescence was due to spontaneous fluorescence emission of HQ addition or IκB–EGFP protein accumulation. The results showed that mock-transfected Huh 7 cells treated with 20 μM or 100 μM HQ had the same fluorescence as untreated cells (Figure 2). The fluorescence intensity of the transfected cells treated with 20 and 100 μM HQ was higher than that of the untreated transfected cells. These results indicated that the change in fluorescence was due to IκB–EGFP protein accumulation but not spontaneous fluorescence of HQ.

**Figure 3:**
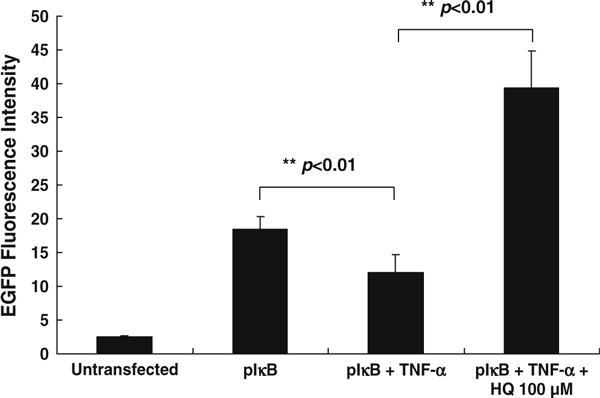
**HQ antagonizes the effect of TNF-α-induced NF-κB production in pIκB–EGFP-transfected 293 cells.** Transfected 293 cells were added either with TNF-α (100 μM) or TNF-α plus HQ (100 μM). After 24-h incubation, the amount of IκB–EGFP proteins (shown as fluorescence intensity) was measured by flow cytometry (results expressed as mean ± standard deviation).

Because the transfection efficiency of Huh 7 cells by liposome method is not high enough, we chose 293 cells to replace Huh 7 cells. Figure 3 shows that 293 transfected cells had higher EGFP fluorescence increase (about 7.3-fold), compared with untransfected 293 cells (Figure 3, columns 1 and 2), than the change in Huh 7 cells (3.2-fold; Figure 2, columns 1 and 4). The difference in fluorescence intensity between Huh 7 and 293 transfectant was due to the variation of transfection efficiency. The pCMV–EGFP plasmid that can constantly express EGFP proteins in cytoplasm has been used to determine the transfection efficiency. The study revealed that the transfection efficiency for Huh 7 and 293 cells was about 25% and 65% (results not shown). TNF-α has been reported as an activator for NF-κB. Therefore, TNF-α shall enhance degradation of IκB–EGFP to result in a decrease in fluorescence. The results showed that TNF-α does decrease the fluorescence of pIκB–EGFP-transfected 293 cells. However, previous treatment of HQ can increase the fluorescence of pIκB–EGFP-transfected 293 cells even in the presence of TNF-α (Figure 3). Therefore, HQ can inhibit the degradation of IκB in 293 cells as well as in Huh 7 cells.

### 3.2 The Activities of Anti-IκB Degradation of the Natural Extracts are Detectable by the IκB–EGFP Drug Discovery System

**Figure 4:**
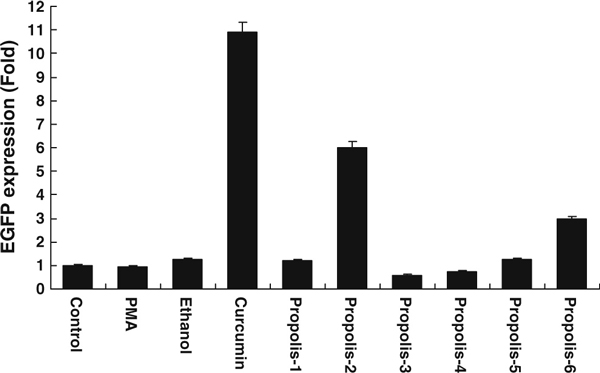
**The screening effect of IκB–EGFP drug discovery system assayed on propolis.** Taiwan propolis (propolis 1 and 2), Brazil red propolis (propolis 3 and 4), and Brazil green propolis (propolis 5 and 6) were dissolved in 80% ethanol and used as test samples to verify the ability of the pIκB–EGFP drug discovery system. pIκB–EGFP-transfected 293 cells were used as a control group; PMA (50 ng/ml) and curcumin were other control groups used for initiating or inhibiting IκB–EGFP degradation. Ethanol (80%) was added into pIκB–EGFP-transfected 293 cells to verify if the fluorescence increase was due to the background effect of solvent used. After 24-h incubation of the propolis, the amount of IκB–EGFP proteins (shown as EGFP expression fold compared to control group) was measured by flow cytometry (results expressed as mean ± standard deviation).

The extracts of propolis were used as samples to verify whether the IκB–EGFP drug discovery system can be responsible for the substances that existed in the natural extracts. The results in Figure 4 showed that propolis 2 and 6 had the activities to suppress the IκB–EGFP degradation, but another propolis did not cause the increase in EGFP fluorescence.

### 3.3 HQ Inhibits the Degradation of IκB by Lowering the Phosphorylation of IκB Protein

**Figure 5:**
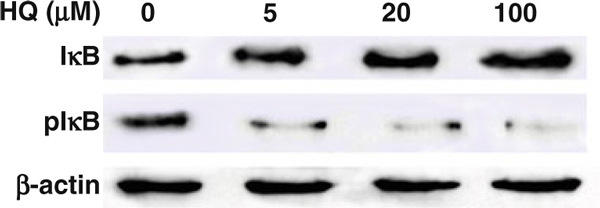
**The mechanism used by HQ prevents the degradation of IκB proteins.** The amount of intracellular unphosphorylated and phosphorylated IκB proteins of 5–100 μM HQ-treated 293 cells was detected by Western blot. The quantity of IκB and phosphorylated IκB proteins was induced by different dosages of HQ.

293 Cells were treated with different dosages of HQ, and the intracellular IκB proteins were detected with dot immunoblotting method. The results revealed that as the concentration of HQ was increased from 5 μM to 100 μM, the amounts of IκB protein also increased in cells (Figure 5a). The phosphorylation of IκB was also determined by anti-phosphorylation IκB antibody. The results showed that HQ could affect the phosphorylation of IκB protein. As the concentrations of HQ were increased, the amounts of phosphorylated IκB protein were decreased. An increase in HQ dosage could inhibit the phosphorylation of IκB protein (Figure 5b).

### 3.4 Temporary Contact of Low Dosage HQ can Inhibit the Cell Adhesion Ability without Cell Lethality

**Figure 6:**
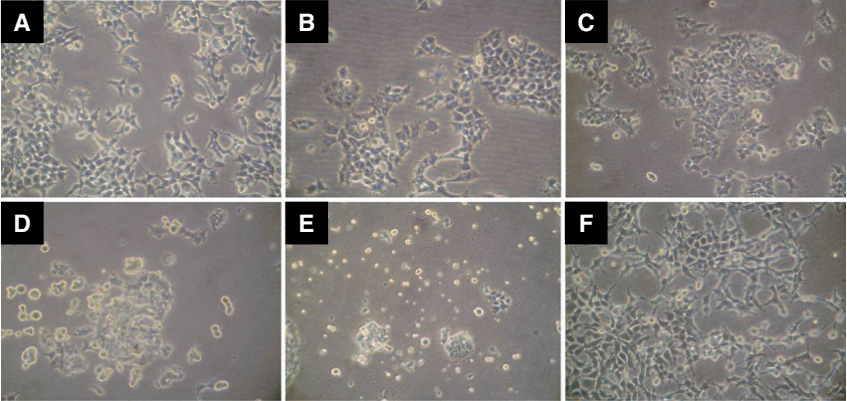
**Cell adhesion ability affected by low dosage of HQ.** 293 Cells were seeded in a six-well plate and incubated with 10 μM HQ. **a**–**e** The cell detachment patterns were observed at 0, 0.5, 1, 2, and 4 h. **f** After this 4-h observation, HQ from 293 cells was removed and seeded again to test the adhesion ability.

293 Cells (2.5 × 10^5^) were exposed to 10 μM HQ. Time-dependent detachment of 293 cells was observed during the short-term contact of HQ (0–4 h) (Figure 6a–e). After HQ exposure, harvesting detached cells and seeding them again found that removing HQ from 293 cells can regain their adhesive ability (Figure 6f).

### 3.5 In Vitro Tumor Cytotoxicity by Long-Term and High-Dose Treatment of HQ

**Figure 7:**
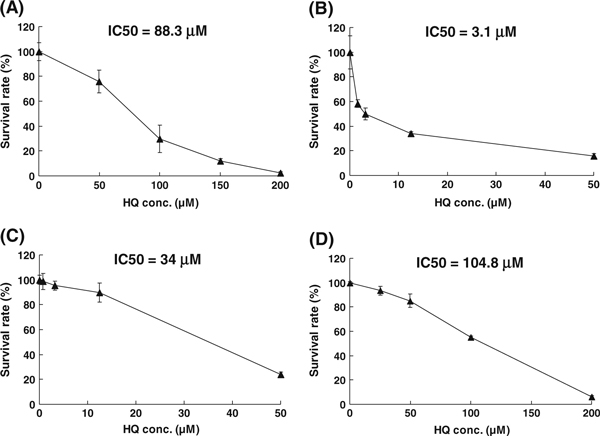
**In vitro tumor cytotoxicity induced by high dosage of HQ.** One hundred microliters of cells suspended in complete medium at a concentration of 2 × 10^4^ cells/ml was seeded in a 96-well plate overnight and treated with different concentrations of HQ. After 24-h incubation, HQ cytotoxicity was determined by MTS assay. **a** COLO320, **b** K-Balb, **c** Hep3B, **d** Daudi cells.

The tumor cytotoxicity induced by HQ was accessed via the cell death rate of COLO320, K-Balb, Hep3B, and Daudi tumor cell lines. A dose-dependent death was observed in all four cell lines. Also, 200 μM HQ caused almost all COLO320 cells to die, and the IC50 was 88.3 μM (Figure 7a). In K-Balb cells, the decrease in cell number was due to increasing concentration of HQ (0–50 μM), and the IC50 was 3.1 μM (Figure 7b). The same situation was observed in the Hep3B cells; 50 μM HQ caused large amounts of cells to detach and die (IC50 = 34 μM) (Figure 7c). In Daudi cells, 0–200 μM of HQ also caused a slow increase in cell death, and IC50 was 104.8 μM (Figure 7d).

## 4 Discussion

Because of rapid degradation of IκB with stimulation, it could be a target to analyze the effect of drugs on the NF–κB signaling pathway [8]. Activation of the NF-κB can be detected by green fluorescent protein-tagged IκB degradation in living cells [9]. In this study, we first used the IκB–EGFP protein as an indicator to screen microbial metabolites and demonstrated that the effective substance selected from the screening system can inhibit activity of NF-κB by inhibition of IκB degradation. Mechanisms for regulation of NF-κB activity comprise inhibiting the activation of IKK complex, inhibiting the phosphorylation of IκB, preventing the degradation of IκB, blocking the migration of NF-κB to nucleus, and decreasing the DNA binding activity of NF-κB. For activation of NF-κB, IκB must be phosphorylated to release itself from the complex of NF-κB and process of degradation. In this study, the results revealed that the degradation of IκB is exactly a good reference to relate the activity of NF-κB. Thus, utilization of IκB degradation as an indicator to judge intracellular NF-κB activity can be established as a drug screen system for selecting microbial metabolites.

The degradation of endogenous IκB regulates NF-κB activation, and IκB can be degraded rapidly within 2 h to prevent the accumulation of IκB. Thus, the stability of cellular IκB is low. In this system, we find that the changes in the amounts of IκB or phosphorylated IκB are similar to endogenous IκB or IκB–EGFP, which is dependent on the stimulation with the NF-κB activator, such as TNF-α or PMA. In addition, the half-life of IκB is greatly shortened to 5–30 min with TNFα-induced IκB degradation. Therefore, we proposed that the endogenous IκB could not influence the expression of IκB–EGFP.

Plasmid pIκB–EGFP was transfected into different cell lines (Huh 7 and 293 cells) to establish cell-based drug screening system, and the results showed that the pIκB–EGFP-transfected 293 cells have better response in fluorescent expression after HQ treatment than Huh7 cell (Figures 2 and 3). Therefore, the sensitivity of IκB degradation-based drug screening system is related to cell type. The cells that have stronger intracellular NF-κB activity may be more proper to develop the screening system.

The most common active form of NF-κB is p50/p65 heterodimer, whereas c-Rel, Rel-B, or p52 also has the ability to form a heterodimer with p50. Except for heterodimers, the homodimers of NF-κB including p50/p50, p52/p52, and RelA/RelA also existed and have activities in cells. These NF-κB dimers have different affinities to NF-κB binding sequence in promoter [10]. In addition, the expressions of identical gene are regulated with different NF-κB dimers in different cells. For example, the vascular cell adhesion molecule-1 (VCAM-1) gene of endothelial cells is regulated by p50/p65; however, the VCAM-1 gene of Hela cell is regulated by p65/p65 but not p50/p65 [11]. It can be concluded that the members of IκB family—IκB-α, IκB-β, IκB-γ, and Bcl-3 proteins—all have the ability to suppress NF-κB translocation into cell nucleus. Whereas different IκB have their own specific target molecules, such as IκB-α that prefers binding to p50/p65 and p65/p65, nevertheless it inefficiently binds to p50/p50 [12]. In addition, IκB-β specifically binds to p65 and c-Rel but not p50, and IκB-γ and Bcl-3 specifically bind to p50 [13]. Thus, different cells have various NF-κB existences and regulate the activity of NF-κB by different IκB members. Because of the widespread importance of NF-κB, it has been difficult to develop NF-κB inhibitors that act specifically in a specific cell to employ in therapy of diseases. Therefore, certain specific IκB inhibitors may be found according to our model presented here and inhibit specific NF-κB activity in certain cells without affecting the NF-κB signal pathway in other cells.

HQ was chosen from this system, and the previous literatures showed that it could decrease the secretion of cytokines [14,15]. HQ exists in extract of tobacco and inhibits human lymphocytes to produce IL-1β, IL-2, and TNF-α [16]. HQ has also been reported to reduce the expression of CD19 by decreasing the intracellular activity of NF-κB, and its mechanism is not due to the inhibition of the DNA binding activity of NF-κB [17]. In this study, we first reveal that HQ inhibits the activity of NF-κB by inhibiting the degradation of IκB.

In this study, we have demonstrated that the plasmid pIκB–EGFP transfectants (Huh 7 and 293 cells) were used as a drug screening system to seek NF-κB inhibitor, and we did obtain HQ of microbial origin, which have been shown to have a significant activity against NF-κB activity, from the screening program. NF-κB target genes have been studied and are involved in immunity, inflammation, cell proliferation, apoptosis, and cell migration [18]. A close connection between inflammation and cancer has also been suspected. NF-κB pathway is also involved in cell adhesion. The cell adhesion molecule, such as integrin, has been correlated with the cell differentiation or adhesion. HQ, a NF-κB inhibitor, can block upstream signaling for both NF-κB activation and cell adhesion. HQ also inhibits NF-κB activation through suppressing integrin expression for cell adhesion [19]. HQ exposure affected cell proliferation and delayed cell growth and attachment in a dose-dependent manner [19]. In addition, HQ diminishes surface levels of CD29 and CD18 and suppresses CD29-mediated cell–cell adhesion in monocyte [20]. Therefore, such downregulation with HQ treatment may be an inhibitory mechanism for cell adhesion. Therefore, the system may provide a good method to develop a new drug for inflammation and cancer therapy (Figure 7).

Propolis was used to determine whether this screening system is sensitive enough to identify the effective substance from a natural extracted mixture. The results showed that propolis increased the fluorescence of pIκB–EGFP-transfected cells compared to negative control groups. Different kinds of propolis were extracted by 80% ethanol, and the results showed that the effective substance for inhibiting the IκB degradation is favorable in ethanol (Figure 4). The previous evidences revealed that the effective substance should be caffeic acid phenethyl ester which is hydrophobic and can inhibit NF-κB activity [21]. In addition, red propolis or green propolis was harvested respectively in winter or summer, and the results also showed different propolises harvested in different seasons have different activities to inhibit the IκB degradation (Figure 4). Propolis is the product after bees harvested the resin from the tree, and different seasons would affect what kind of tree resin was harvested by the bee. Thus, the propolises have different activities to IκB degradation that is reasonable. This study showed that this assay could determine the activity of natural product for IκB degradation.

In this study, an assay was established to monitor the activities of substances for IκB degradation in the cell. The results showed that the assay could be applied to pure chemical and natural mixture. After selection by this assay, the chosen candidate exactly has the ability to cause the change in the NF-κB activity via regulating the IκB and further regulate the expression related with NF-κB. Therefore, the system may provide a good method to develop a new drug for certain diseases that are related to NF-κB activities such as inflammation and cancer.
